# Understanding Molecular Mechanisms and Identifying Key Processes in Chronic Radiation Cystitis

**DOI:** 10.3390/ijms23031836

**Published:** 2022-02-06

**Authors:** Clément Brossard, Anne-Charlotte Lefranc, Jean-Marc Simon, Marc Benderitter, Fabien Milliat, Alain Chapel

**Affiliations:** 1 Service de Recherche en Radiobiologie et en Médecine Régénérative (SERAMED), Laboratoire de Radiobiologie des Expositions Médicales (LRMED), Institut de Radioprotection et de Sûreté Nucléaire (IRSN), 31 Avenue de la Division Leclerc, Fontenay-aux-Roses, 92260 Paris, France; clement.brossard@irsn.fr (C.B.); charlotte1810.lefranc@gmail.com (A.-C.L.); marc.benderitter@irsn.fr (M.B.); fabien.milliat@irsn.fr (F.M.); 2 APHP, Hôpital Universitaire Pitié-Salpêtrière, Radiotherapy-Oncology Department, 47-83 Boulevard de l’Hôpital, CEDEX 13, 75651 Paris, France; jean-marc.simon@ap-hp.fr

**Keywords:** chronic radiation cystitis, radiotherapy, cancer, inflammation, vascular lesions, fibrosis, molecular mechanism

## Abstract

Chronic radiation cystitis (CRC) is a consequence of pelvic radiotherapy and affects 5–10% of patients. The pathology of CRC is without curative treatment and is characterized by incontinence, pelvic pain and hematuria, which severely degrades patients’ quality of life. Current management strategies rely primarily on symptomatic measures and have certain limitations. Thanks to a better understanding of the pathophysiology of radiation cystitis, studies targeting key manifestations such as inflammation, neovascularization and cell atrophy have emerged and are promising avenues for future treatment. However, the mechanisms of CRC are still better described in animal models than in human models. Preclinical studies conducted to elucidate the pathophysiology of CRC use distinct models and are most often limited to specific processes, such as fibrosis, vascular damage and inflammation. This review presents a synthesis of experimental studies aimed at improving our understanding of the molecular mechanisms at play and identifying key processes in CRC.

## 1. Introduction

With more than 19 million new cases and 9.9 million deaths in 2020, cancer is the second most common cause of death worldwide. Abdomino-pelvic cancers account for approximately 25% of all cancers, with the most common being cancer of the prostate, colon and rectum [1]. Radiotherapy is used in 50% of cancer treatments [2] and is one of the most effective cancer treatments. Currently, 5% of patients will develop pelvic complications. However, advances in radiation therapy improve outcomes and enhance survival. In the bladder, uterus, and prostate cancer, the organs at risk following radiotherapy are the rectum and the bladder. The most common symptoms are proctopathy (5–20%) and radiation-induced cystitis (3.5%) [3]. Radiation-induced cystitis tends to be clinically severe and can cause extreme pain, hematuria, and irritative voiding symptoms. There is no definitive time frame that constitutes the “at risk” window for radiation-induced cystitis. 

Patients can experience a wide range of symptoms, from mild pelvic pain to life-threatening hemorrhagic cystitis weeks, months, or even years after treatment. To date, there is no way to predict which subset of radiation treatment patients will experience this potentially devastating complication. In its severe form, pain and hemorrhagic cystitis impair patient quality of life, prolong hospitalization and may be life-threatening complications [4]. Moreover, despite recovery, patients who have experienced radiation-induced hemorrhagic cystitis have lower overall and event-free survival compared to patients who have not experienced hemorrhagic cystitis [5]. Treatment is limited to managing the symptoms and is not always effective because the causes may be multiple. The etiology of the disease requires in-depth knowledge of the mechanisms involved, which remain poorly understood [6,7]. It is, therefore necessary to develop a thorough understanding of these mechanisms so that we can propose alternative therapies. Based on molecular knowledge from preclinical studies, we present a synthesis of the mechanisms involved in radiation-induced cystitis in order to identify key effectors.

## 2. Radiosensitivity of the Bladder

The goal of radiotherapy is to eliminate the tumor while preserving the surrounding healthy organs, called the at-risk organs. The total dose that at-risk organs can tolerate depends on the sensitivity of each organ to irradiation, and on the fraction of the organ irradiated. The risk to an organ depends on its sensitivity to irradiation, the dose and the exposed volume (depending on the proximity of the targeted tumor, the delimitation of the tumor), the prescribed fractionation and the type and energy of irradiation used (photon, neutron, electron, proton, carbon, etc.). The bladder is an organ at risk in all radiation treatments for pelvic cancers, particularly prostate cancer (Table 1).

**Figure 1:**
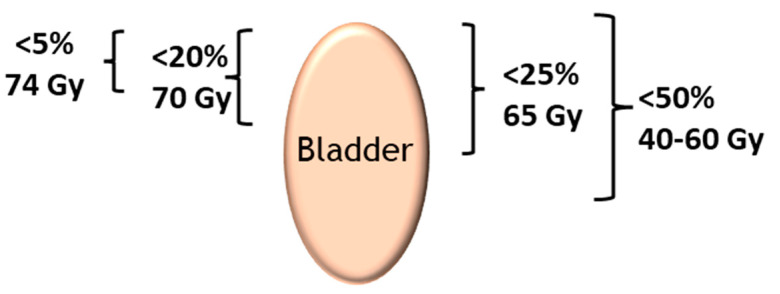
Bladder dose constraints in conformal radiotherapy as a function of the percentage of whole bladder exposed. Establishing the dose constraints for the bladder for a given surface area makes it possible to assess a dose-volume effect on urinary toxicity. The tolerance dose is often expressed as follows: Vx < Y%, which means that the dose X Gy must not be delivered in more than Y% of the V volume of the organ at risk [9,10].

The long-term complication rate is influenced by the three key components of the irradiation protocol, as follows:

1. The exposed bladder volume and surface area—if involved, the trigone is more problematic than the bladder dome.
2. The dose rate—a rate below 0.8 Gy/h reduces the risk of cystitis and the dose of the daily fraction of irradiation. Doses higher than 2 Gy per fraction increase the risk of delayed sequelae.
3. The total dose—toxicity is higher when the overall dose is higher than 60 Gy to the bladder; conformal beam therapy provides higher doses to the target tissue while retaining a lower total dose in the bladder. (Figure 1) [8].

## 3. Incidence of CRC

**Figure 2:**
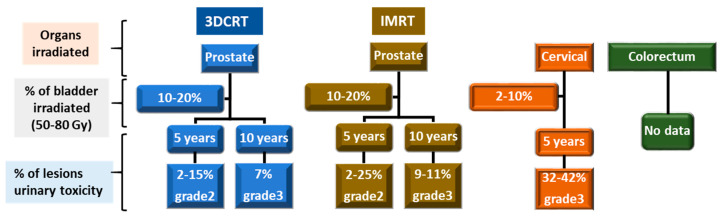
CRC symptoms are a function of the dose and the surface area of the bladder irradiated. This Figure summarizes our knowledge regarding chronic lesions of the bladder for 3DCRT and IMRT for urinary toxicity. The percentage of grade 2 or 3 lesions for a dose range from 50 to 80 Gy is indicated for 5 and 10 years after radiotherapy.

The risk of urinary toxicity increases linearly over time but without a plateau [11,12,13,14]. The percentage of lesions for urinary toxicity in the case of three-dimensional conformal radiation therapy (3DCRT) and intensity modulated radiotherapy (IMRT) for a total dose from 50 to 80 Gy and for a volume of bladder irradiated from 2 to 20% is presented in Figure 2.

However, it is difficult to determine the % of CRC and its symptoms. Indeed, the large number of irradiation techniques, as well as the related radiotherapy protocols, means that the percentages for each symptom can vary greatly. In addition, there are few studies on these subjects and often little or no detail is provided, making it difficult to synthesize the data. Here, we have given an overview of the frequency of the different symptoms depending on the organ treated and the time after radiotherapy.

## 4. Pathophysiology of CRC in Patients

The acute phase of radiation cystitis mostly occurs during radiation treatment. The early effects are damage to the urothelium and microvasculature. Microvascularization damage induces the development of edema in the bladder wall [15]. Edema of the mucosa, submucosa and lamina propria develops, accompanied by acute inflammation. At the level of the cells of the urothelium, nuclear pycnosis and hyperchromatic nuclear coloration are observed; the nuclei may also be enlarged and atypical. Tight junctions and the polysaccharide layer are disrupted, allowing hypertonic urine and isotonic tissue to come into contact, resulting in tissue inflammation and early symptomatic acute radiation cystitis.

Damage to the mucosal wall may be caused by local necrosis of the urothelial cells. These lesions may appear in the first few months after irradiation or appear more than a year after exposure when the chronic phase is initiated. Vascular endothelial cells are thought to be the primary target cells for bladder injury after radiation, with late bladder fibrosis occurring because of vascular ischemia of the bladder wall. Vascular endothelial cell edema is evident at three months and endothelial cell proliferation is evident at six months. The main features are vascular endothelial cell edema, endothelial cell proliferation, perivascular fibrosis. Vascular ischemia, edema, and cell destruction cause the replacement of bladder smooth muscle fibers by fibroblasts and increased collagen deposition. The foremost processes are smooth muscle edema, smooth muscle replacement by fibroblasts, increased collagen deposition, and vascular ischemia [16,17].

The impact is that urinary frequency, dysuria and lower bladder capacity increase [18]. Edemas of the mucosa, submucosa and lamina propria potentiate these effects. Hematuria may develop in relation to petechiae into the lamina propria and submucosa. It should be noted that the appearance and intensity of acute lesions are not related to the severity observed at the chronic level [19,20].

The chronic effects of radiation cystitis differ from the acute effects. Chronic radiation cystitis can develop between 6 months and 20 years after radiation therapy. Factors related to the patient, as well as to the tumor and treatment, increase the risk of developing chronic radiation cystitis. Diabetes, hypertension and previous abdominal surgery are risk factors. Surgery, postoperative sequelae, and chemotherapy can contribute to the development of chronic radiation cystitis. The volume of tissue irradiated, the dose to the bladder as well as concomitant treatments and individual radiosensitivity are aggravating factors. The symptoms of the chronic phase are the result of a combination of processes that range from the deregulation of urothelium homeostasis, hyperactivation of the endothelium, inflammation, hypoxia, oxidative stress, defect in neuroregulation and fibrosis. These processes potentiate each other, allowing the progressive worsening of the CRC. Ischemia, fibrosis and neuroinflammation appear to be the main factors responsible for the symptoms of CRC; however, they are permanently potentiated by all the processes stated above. Consequently, some symptoms of CRC appear years after radiotherapy, the most obvious of which are bladder contraction, ulcer formation, fistula, and bladder dysfunction. Therefore, the pathology of CRC should include the following urinary symptoms: frequency, urgency, dysuria, hematuria, pain, degradation of quality of life [21]. Cytoscopy can be used to classify the three main forms of CRC [22]: a predominantly inflammatory form with edema, mucosal pallor and possible ulceration, a predominantly bleeding form with friability, spontaneous hemorrhage and telangiectasia, and a mixed form with features of inflammatory CRC and hemorrhagic CRC [19,22,23,24,25].

## 5. Grading CRC

Lesions can range from a simple inflammatory reaction to almost complete loss of bladder function. The main clinical symptoms are macroscopic hematuria accompanied by pain and pollakiuria. Different scales for rating the severity of CRC exist. The European Organization for Research and Treatment of Cancer and Radiation Oncology Therapy Group (RTOG/EORTC) scale for calculating radiation toxicity of exposed organs is the most widely used. Another commonly used scale is the LENT-SOMA scale, which stands for Late Effects Normal Tissue (LENT)-Subjective Objective Management and Analytic (SOMA) assessment. Each known individual organ or tissue has its own LENT-SOMA scale [26]. The LENT-SOMA scale is a comprehensive system and provides a great deal of information but can be difficult to implement outside clinical trials (Table 2). Several classification systems for the severity of hemorrhagic cystitis have been used in published reports and are presented in Table 2 [27,28,29,30,31,32]. Today, the most widely used system is the CTCAE v5.0 system [33]. All these scales are mainly based on the degrees of hematuria (microscopic to macroscopic) and their frequency as well as patients’ bladder capacity. The classifications do not take into account the degradation in patients’ quality of life due to CRC, whereas the bladder seems to tolerate high doses of ionizing radiation, as demonstrated by the long-term follow-ups of bladder cancers treated with radiotherapy by Lars Henningsohn et al. [34]. In this study, the symptoms are studied one by one, which makes it possible to advance and understand the underlying pathophysiology. This study has the great merit of correlating symptoms and pathophysiology with the long-term quality of life of patients after radiotherapy, which is too rarely done. This study shows that it is essential to take account of the patients’ distress, their pain, the degradation of their quality of life and their sexual comportment in pathophysiological investigations and in the classifications.

## 6. Clinical Management of Radiation Cystitis

**Figure 3:**
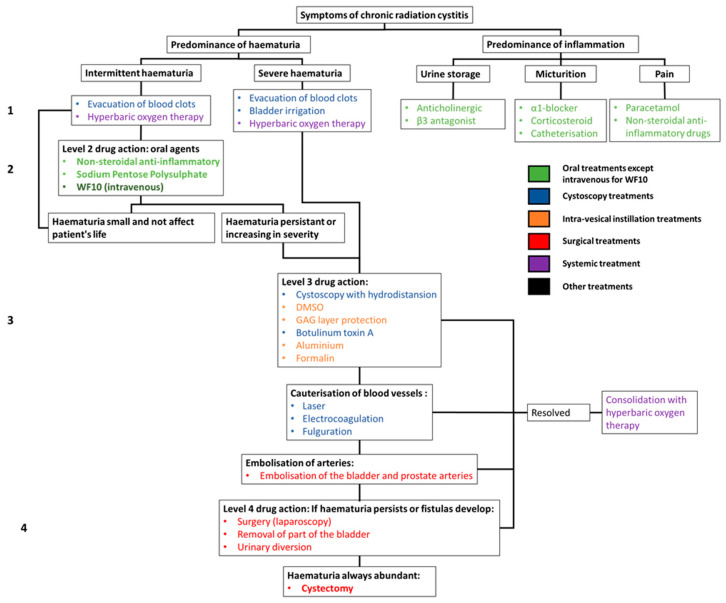
Decision tree for the management of chronic radiation cystitis and its treatment.

Conventional treatments focus on reducing the episodes and intensity of hematuria and inflammation (Figure 3) [35]. In the case of inflammatory CRC, problems mainly occur during the urine storage phase, urination and/or chronic pain. Treatments for pain in the voiding phase are α1-adrenoceptor antagonists (α1-blockers), corticosteroids, as well as catheterization to avoid pain. General pain can be treated with paracetamol or non-steroidal anti-inflammatory drugs (NSAIDs) [22,36].

In the case of hemorrhagic CRC, the first step is to establish the origin of the bleeding. To do this, a cystoscopy is performed to check the condition of the bladder wall and that of the blood vessels. The treatment of hemorrhagic CRC will depend on the intensity of the hematuria.

First-line treatments for hemorrhagic CRC can be used for grade 1 on the LENT-SOMA scale. These include blood clot evacuation and hyperbaric oxygen therapy. In cases where the hematuria is sudden and severe, blood transfusion may be required, followed by hyperbaric oxygen therapy [37].

For intermittent but persistent hematuria, oral treatments such as nonsteroidal anti-inflammatory drugs (NSAIDs) and glycosaminoglycan (GAG) protectors (WF10 intravenous and sodium polysulphate) are prescribed.

If the hematuria continues to worsen (above grade 2 on the LENT-SOMA scale) more severe treatments are required. These include cystoscopy with hydrodistention or botulinum toxin injection. Intravesical instillations are also used. These include dimethyl sulfoxide (DMSO) or GAG layer protectors (sodium pentose polysulphate, heparin, etc.) and, as a last resort, aluminum and formalin.

If the CRC continues to worsen, it is possible to cauterize the blood vessels (via laser, fulguration, or electrocoagulation) and then embolize the bladder and prostate arteries. If this is not enough, it is possible to perform laparoscopy, partial bladder removal or urinary diversion and, as a last resort, a cystectomy [6,7,22,38,39,40,41]. The effect of treatment on the parameters of pollakiuria, dysuria and pain have not been extensively studied, the result of only a partial evaluation of the effectiveness of the therapies tested. Various side effects of these therapies are known, including risk of intoxication, bladder rupture, kidney failure, fibrosis and even death. According to the literature, these techniques and methods may show some slight decrease in hematuria grades [5,42,43,44,45,46,47,48,49,50,51,52,53,54,55]. The results may be short-term effects with significant side effects such as intense pain, to the point of having to interrupt treatment [40]. Hyperbaric oxygen therapy may be the method that seems to show the highest efficacy (75% improvement within six months) with the least side effects, which can include barotraumatic otitis, transient visual disturbances and paresthesia of the fingers. However, hyperbaric oxygen treatment is constraining and expensive, requiring 30–40 sessions of 80–90 min over two months, plus the effects diminish with time and may recur a few years after treatment, up to 40%, especially in the most serious cases [37,56] (Figure 3).

## 7. Mechanisms of CRC: Lesson from Preclinical Studies

**Figure 4:**
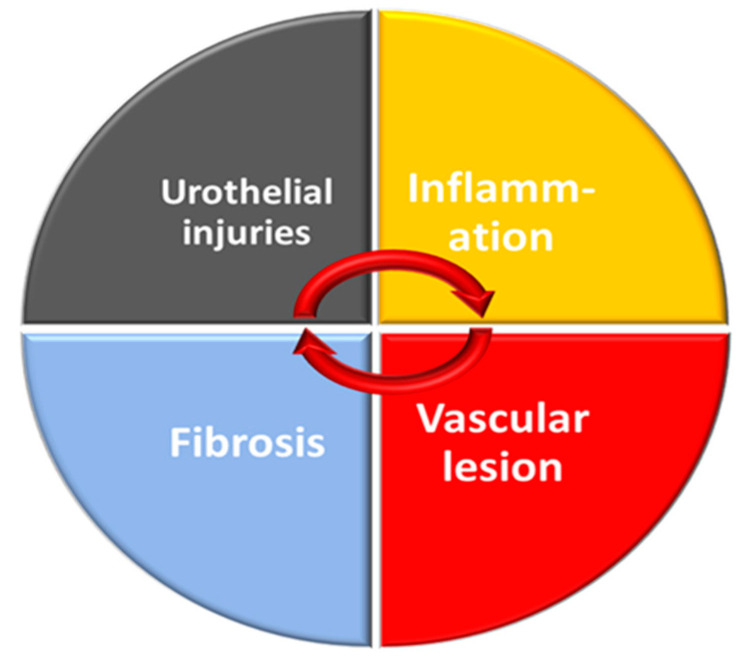
Relationship between the main mechanisms involved in CRC.

The chronicity of the effects of radiation therapy involves mechanisms common to most healthy tissue exposed to radiation. These mechanisms are also found for CRC. The chronicity of radiation effects on healthy tissue is associated with endothelial damage with late vascular repercussions and disruption of angiogenesis. This phenomenon induces a chronic inflammation. In the longer term, the implementation of these mechanisms leads to fibrosis and disorganization of the muscle layers. As detailed below, analysis of preclinical data confirms that the mechanisms are similar although they are less detailed in CRC. The pathology of CRC shows that there are two predominant forms, namely an inflammatory form and a hemorrhagic form, which can be combined (as described above). Based on this observation, there seem to be two major mechanisms of CRC: chronic inflammation and vascular damage. Given that inflammation is linked to pain, it seems likely that there is a role for neuro-inflammation in this process, inducing a lack of neurogenic control. (Figure 4).

However, clinical trials, as well documented as they are, unfortunately do not explain the mechanisms of CRC. On the other hand, preclinical studies provide more information regarding homogeneous animal cohorts in term of doses as well as irradiation protocols, combined with a precise monitoring of the animals for long-term follow-up. However, one must keep in mind the limitations of animal models when extrapolating mechanisms to human pathology. To illustrate this, in humans, hemorrhages are a major component of CRC, but they appear very sparsely in animals. A review of original articles was performed using PubMed from 1950 to 2020. We used the following search terms: Chronic radiation cystitis, radiation cystitis, radiation-induced cystitis, radiation-induced hemorrhagic cystitis, radiation damage bladder. We retained only articles discussing mechanisms, and excluded articles referring to treatments. Then we selected only those articles that study the chronic phase. In all, eighteen articles have been selected, as summarized in Table 3.

### 7.1. Urothelium: Unknown Actor but May Be the Key Element in Inflammation: A Paradigm Shift?

We decided to approach CRC through the loss of impermeability of the urothelium, although the endothelium is described as the key element in chronic radiation sequelae on healthy tissue. The loss of impermeability of the urothelium is the loss of a protective barrier of the bladder. This is the first element allowing deterioration of the underlying tissues. Maintaining the impermeability defect over time due to a defective repair may enable implementation of the mechanisms observed during the chronic phase. Many agents can maintain these lesions of the urothelium in patients and could be facilitating agents for the establishment of CRC. The observation that visible lesions on the urothelium occur early in CRC and may be the result of improper acute phase repair confirms that the urothelium may be the initiating point of CRC. This lack of repair can lead to inflammation, which can participate in vascular damage and thus lead to the development of CRC.

**Figure 5:**
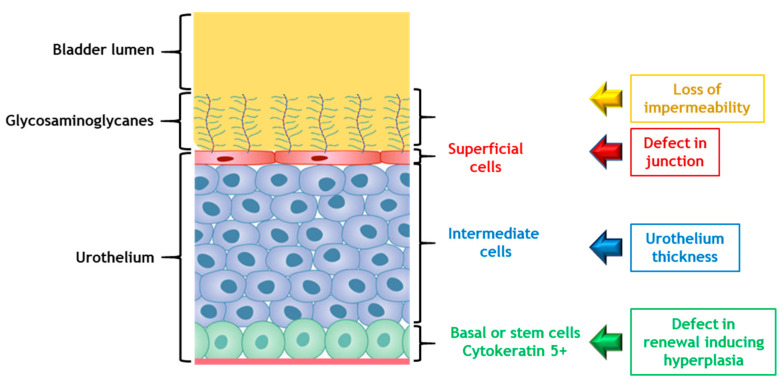
Abnormalities in urothelium regeneration and loss of impermeability.

Damage to the visible structure of the urothelium occurs mainly with hyperplasia. The urothelium also undergoes molecular changes, with a decrease in tight junctions such as uroplakine III or E-cadherin inducing an increase in bladder permeability. In the first phase of CRC, a decrease in the number of superficial cells was observed [20,68]. In the late phase, the main feature in studies is that the urothelium presented lesions at the structural level. In the studies of Zwaans et al. and Jaal et al., a decrease in the expression of uroplakine 3 was detected in superficial cells as well as in the expression of E-cadherin. These decreases induced a diminution in bladder tightness. Infiltration of urine into the tissues maintained inflammation and urothelium lesions accompanied by hyperplasia and edemas [20,68]. Hyperplasia remained the most frequent phenomenon [20,64,68,72]. In preclinical studies, hyperplasia, erosion of the urothelium, atrophy and cellular edema, especially in the basal cells, are common mechanisms found for several animal species and irradiation configurations. Edema and hyperplasia were also described in CRC patients [72]. The integrity of the urothelium is maintained through a complex process of migration, proliferation and differentiation of stem cells. All preclinical studies confirm the damage to the urothelium resulting in edema, hyperplasia and a decrease in the thickness of the urothelium. There seem to be abnormalities in the regeneration of this tissue, which could be related to a deregulation of the proliferation and differentiation of stem cells of the urothelium. Accelerated proliferation can occur in various bladder pathologies. For example, during the use of chemical agents such as cyclophosphamide that damage the umbrella cell layer [73,74]. Slow urothelial cell turnover and differentiation can be influenced by exogenous factors, including epithelial growth factor (EGF), platelet-derived growth factor (PDGF), peroxisome proliferator-activated receptor (PPAR) and other molecules such as retinoic acid [75,76,77]. We can hypothesize that during CRC there is a deregulation of urothelial stem cells and that factors such as EGF, PDGF and PPAR could contribute to this deregulation (Figure 5).

### 7.2. Vascular Lesions Are the Checkpoint in Chronicity Development

#### 7.2.1. Vascular Damage Implicated in Late Radiation Injury

**Figure 6:**
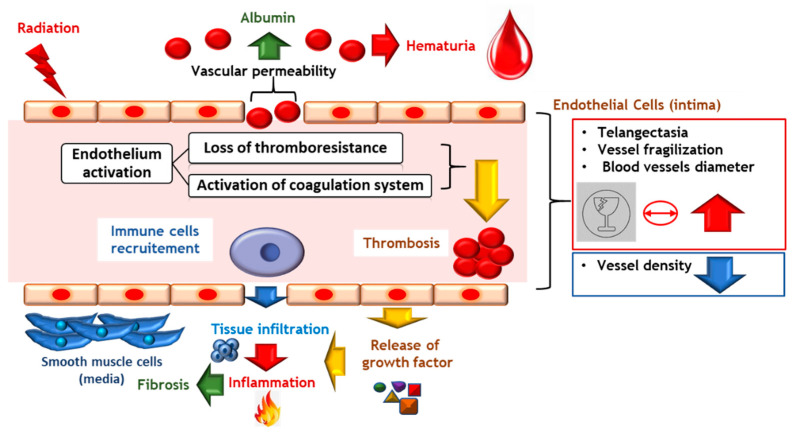
Endothelial activation induces vascular permeability and tissue infiltration and stimulates a pro-thrombotic and pro-inflammatory phenotype that leads to thrombosis and leukocyte recruitment.

In survivors of radiation-treated cancers, vascular damage contributes to the initiation and subsequent progression of radiation-induced tissue damage [78]. Ionizing radiation, through its effects on the endothelium, mediates initiation and progression of radiation-induced damage to healthy tissue. Endothelial apoptosis increases vascular permeability, endothelial activation and recruitment of inflammatory cells, and activation of the coagulation system, contributing to the progression of radiation-induced tissue damage [79]. Endothelial cells play a key role in the control of vascular tonicity by secreting molecules that act on vascular smooth muscle cells [80]. The main effects are radiation-induced cell death, loss of thromboresistance and secretion of soluble factors, such as cytokines or growth factors. Thrombomodulin deficiency is demonstrated after irradiation and is chronically perpetuated in rats and humans [81,82]. The mechanisms responsible for the decrease in thrombomodulin expression are related both to the direct effects of irradiation through the production of reactive oxygen species and to indirect secondary effects related to radiation-induced inflammation [83]. The loss of endothelial thromboresistance following ionizing stress results from the stimulation of fibrinogenesis and a decrease in fibrinolysis associated with overexpression of tissue factor [84], von Willebrand factor [85] and a decrease in the expression of prostacyclin [86] and thrombomodulin [87]. Activation of the coagulation system is one of the physiological processes stimulated after irradiation. The endothelium is in a chronic procoagulant state, which may contribute to the long-term persistence of the deleterious effects of irradiation. A correlation exists between vascular thickening and radiation-induced tissue damage [80]. The late effects are tissue hypoxia related to vascular ischemia and chronicity of endothelial dysfunction (Figure 6).

#### 7.2.2. Vascular Lesions: Key Factor in the Onset of CRC

As described for preclinical studies, it appears that in CRC, as in other chronic radiation pathologies, early events in endothelial cells contribute to the progression of radiation-induced vascular and tissue damage. The late presence of vascular lesions might be explained by the slow renewal of the endothelium [58]. According to Stewart et al., irradiation with a single dose starting at 25 Gy induced compensatory proliferation of the endothelium, but the response did not initiate until several months later. Endothelial cell proliferation started from six months and continued until 22 months after irradiation (end of study). Irradiation seems to accelerate the rate of proliferation of endothelial cells. According to the authors, the rate of endothelial cell renewal increased from one week to 6 months after irradiation, whereas the rate of endothelial cell renewal is one year for non-irradiated controls. Furthermore the increase in proliferative activity was dose-dependent at 22 months after irradiation [58,61].

Irradiation doses, corresponding to a therapeutic dose or following irradiation accidents, have deleterious effects on the neovascularization process. In preclinical models of CRC, vascular lesions, joined with telangiectasias and the presence of albumin, have led to the activation of macrophages and mast cells that released large quantities of pro-inflammatory molecules, including tryptase, which maintained and amplified vascular lesions and fibrosis [88,89]. These alterations, might, as they worsen, lead to microscopic then macroscopic hematuria visible under cystoscopy in patients with CRC [90,91]. Vascular lesions are the key focus of CRC. Vascular lesions are due to irradiation and appear after the acute phase due to slow endothelial cell turnover. These lesions appear in the chronic phase, inducing inflammation and neo-vascularization. In patients, an increase in the Placental Growth Factor protein (PGF) was measured in the urine at an early stage up to one year after irradiation. This molecule promotes angiogenesis by binding to the vascular endothelial growth factor (VEGF) receptor, VEGFR2. VEGFα, another major player in angiogenesis, is an increased protein concentration in the urine of patients with hemorrhagic cystitis.

Soler et al. observed a decrease in the number of blood vessels in irradiated rats (20 Gy) up to 90 days post-irradiation (end of study). Despite this decrease there was no change in gene expression of VEGF alpha in the bladder wall, no hypoxia (based on the level of hypoxia-inducible factor-1 (HIF-1) α expression). The loss of vessel density was greater in the cranial part of the bladder (the dome) [70]. Jaal et al. measured an increase in blood vessel surface area throughout the late phase for up to one year [67]. These two results do not contradict each other. Soler’s study measured the number of vessels, while Jaal’s study quantified the total surface area of the vessels, not their number. Therefore, there was a decrease in the number of vessels, but these vessels were wider, which was in agreement with the telangiectasias visible in the Zwaans [15,20] and Kohler studies [64]. 

The vascular lesions are a common mechanism found in these studies independently of animal models and the irradiation configuration. Telangiectasia is the most commonly observed phenomenon, which is not the case for hematuria, as opposed to what is observed among patients. These lesions are one of the first visible phenomena of CRC. Telangiectasias are accompanied by an increase in the permeability of the small blood vessels. All these changes cause the small blood vessels to deteriorate, enabling their rupture during bladder contraction and distention. Original studies by Zwaans et al. indicated significant blood vessel alteration with pale avascular lesions, hemorrhagic lesions and telangiectasias on the largest blood vessels one year after irradiation at 40 Gy. Telangiectasia is defined as permanent dilatation with thickening of the blood vessel wall. It should be pointed out that lesions were visible in all strains of mice tested [15]. Telangiectasias were also noticeable in histology 130 days after irradiation (about 4 months) in the submucosa from 20 Gy in CH3/HeN mice [20]. Telangiectasia was similarly measured in rabbits in the study of Kohler et al. two years after irradiation from 33 Gy and found to be proportional to the irradiation dose. Telangiectasias mostly affected the caudal part of the bladder, although without being statistically significant [64]. The telangiectasias of the small blood vessels make them more fragile with regard to the contractions and dilations undergone by the bladder. Jaal et al. [69] revealed an increase in the permeability of the blood vessels characterized by the presence of albumin around these vessels. The presence of albumin was detected throughout the late phase from 90 days to 360 days. However, albumin was not observed around the large blood vessels.

As opposed to CRC human pathology, the majority of preclinical studies did not show hematuria. Only the model of Kohler et al. showed microhematuria. This presence of hematuria might be explained by higher doses compared to the other models studying vascular lesions. Another explanation could be the longer study time—two years—compared to one year in the studies of Zwaans and Jaal [20,68,69,92]. This observation regarding the absence of hematuria in animal models of CRC leads us to question the limitations of preclinical studies in explaining the mechanisms of CRC in humans.

### 7.3. Inflammation Is a Major Tissue Response to Irradiation, Inducing Chronic Tissue Damage

Inflammation is a complex mechanism that results in damage to the vascular system, induces migration of leukocytes into the irradiated area and secretion of multiple immune system agents [92,93]. Irradiation modifies the functions of immune cells. The multiplication of macrophages and T cells leads to the production of inflammatory mediators, such as Nuclear Factor NFκB (nuclear factor-kappa B) and SMAD2/3 (mothers against decapentaplegic), and cytokines, including (interleukin) IL-1, IL-2, IL6, IL-8, IL-33, TNFα (tumor necrosis factor-α), TGF-β (transforming growth factor-β), and IFNγ (interferon γ), [94]. A close association exists between chronic inflammation and oxidative damage following irradiation [95]. The high amount of inflammatory mediators is combined with the production of prostaglandins and free radicals, which include reactive oxygen species (ROS) and nitric oxide (NO) [96]. The persistent exchange between the immune system and the radiation-damaged tissues in turn amplifies the inflammatory loops mobilizing more inflammatory cells.

The lack of data in preclinical studies on chronic inflammation in CRC, the main inflammatory cells involved, and the regulatory processes are real obstacles to understanding the role of inflammation in CRC pathology. Only the presence of mast cells at the initial phase of CRC has been demonstrated. Compared to similar pathologies, such as interstitial cystitis, we may suggest that degradation of the urothelium leads to the release of histamine, IL-6, VEGF and tryptase [87]. The release of these factors could activate mast cells, which may favor chronic inflammation in CRC. In preclinical studies, the mechanisms of chronic inflammation have only partially been studied in CRC. However, at cellular level, several studies revealed an increasing number of pro-inflammatory cells. Zwaans et al. demonstrated a slight increase in the number of mast cells 130 days after irradiation into the submucosa. [20]. However, mast cells appeared at the end of the early phase [52] and remained in the late phase into the submucosa [69]. No other studies have investigated pro-inflammatory mechanisms in the chronic phase. However, Jaal et al. demonstrated that COX-2 (Cyclooxygenase-2), which had a role in inflammation, was not expressed in late-phase tissue [67]. The presence of mast cells at the end of the early phase and at the initiation of the late phase supported chronic inflammation. Mast cells actively participated in the production of pro-inflammatory cytokines and in vascular injury.

Chronic inflammation plays a pivotal factor in the acquisition and evolution of CRC, as well as in many chronic diseases. What is not described in CRC and yet is the main feature of chronic inflammation is the prominent presence of macrophages and lymphocytes, including B cells, plasma cells and T cells. Therefore, it is possible that the chronic inflammation that characterizes CRC might include macrophages and T cells, in addition to the mast cells described in the clinical trials.

#### Are Neuro-Inflammation and Lack of Neurogenic Control the Cause of Spasms and Pain?

The nervous compartment has a very important role during urination, ordering the detrusor muscles to contract to empty the bladder. Radiation cystitis is associated with a functional disorder between the nervous system and the bladder generating random and incomplete contractions (phenomenon of the overactive bladder), which contributes to incontinence but also in the pain expressed by patients. In interstitial cystitis, it has been shown that there is significant neuro-inflammation involving mast cells via different signals (nerve growth factor (NGF), substance P (SP)) and leading to the “suffering” of nerve fibers [88,97,98,99,100,101,102]. In the case of radiation cystitis, such neuro-inflammation could also play a prominent role, especially in its initiation and development. Studies by Durand et al. have shown that colon irradiation induces visceral hypersensitivity, partly by acting on mast cell activation and their interaction with nerve fibers within the colon [103]. Based on all these arguments, we hypothesize that this neuro-inflammation could be one of the mechanisms of action generating pain and spasm during CRC. It is important to recognize that a key point that is missing from our understanding of this pathology is that radiation-induced cystitis should not be viewed solely as a chronic inflammation of the bladder wall. As suggested by Giglio et al. (2018), it could be a lack of neurogenic control inducing a loss of functional control [104]. Irradiation affects several levels of neural control of the bladder, notably by sensitizing the micturition reflex. The efferent part of the neuronal control of bladder function would be depressed by irradiation of the bladder, while the afferent part of the neuronal control would be sensitized. The neural control of the irradiated bladder would change from a cholinergic regulation modulated by the interstitial cells to a purinergic regulation. Regulation by these interstitial cells on the micturition reflex would lead to increased sensitivity of the inputs of afferent neurons after irradiation of the bladder [104]. These interstitial cells could be mast cells [105].

### 7.4. Fibrosis: Key Point in Maintaining the CRC

In irradiated tissue, fibrosis is closely associated and overlaps with inflammation. In all the models studying fibrosis, an increase in collagen density was observed, together with infiltration of extracellular matrix (ECM) in the muscular part, within two to six months after irradiation and for doses of 20 to 40 Gy. Fibrosis is a key issue in maintaining CRC. Tissue rigidification will contribute to bladder contractility problems. Both sets of factors promote vascular damage, notably by causing the rupture of these vessels, creating hematuria and generating inflammation again. TGF-β1 is a major contributor to fibrosis in chronic radiation cystitis. Kraft’s study [66] shows that TGF-β 1 is strongly expressed in the urothelium and submucosa, and diffusely in muscle layers. Overall, the studies revealed that the release of TGF-β 1 is correlated with the irradiation dose and post-irradiation time. The increase in collagen has been studied in several models. The study published by Kraft et al. showed an increase in the expression of collagens I and III in tissues and mainly in the submucosa and muscle from 180 days (six months) after irradiation [66]. However, the collagen I/III ratio differed between mouse strains [20]. The study performed with the C3H/HeN Af- nu+ strain showed a decrease in this ratio while the C3H/Neu strain showed an increase. Type I collagen is important for the healing, elasticity and maintenance of connective tissue. Collagen III is the major constituent of the extracellular matrix. Change in ratio could indicate scar tissue. In the model of Zwaans et al., an increase in the density of collagen fibers was evidenced but not in their length or diameter in mice one year after irradiation for a single dose of 40 Gy. An increase in collagen-positive cells I and III was observed in C57BL/6 mice but not in CH3 and BALB/c mice. An important point to emphasize is that the genetic parameter of mice strain plays an important role in response to radio-induced fibrosis [15,20]. Fibrosis was measured in CH3/HeN mice 130 days after irradiation (approximately four months) for a single dose of irradiation of 20 Gy [20]. In the study by Soler et al., an increase in the collagen/muscle ratio was demonstrated at three months post-irradiation in Lewis rats for a single dose of 20 Gy [70]. Histological sections revealed an infiltration of the extra cellular matrix between muscle fibers. Moreover, this increase was homogeneous in the three parts of the bladder (dome, body and trigone) [70]. In the model used by Ikeda et al., fibrosis was also visible 60 days after irradiation [71]. Finally, the fibrosis process is well described in CRC and the pathway is identified.

### 7.5. The Impaired Muscle Induces Contractility Deficit, and Increases Frequency of Miction as Well as Decreasing the Volume of Miction

**Figure 7:**
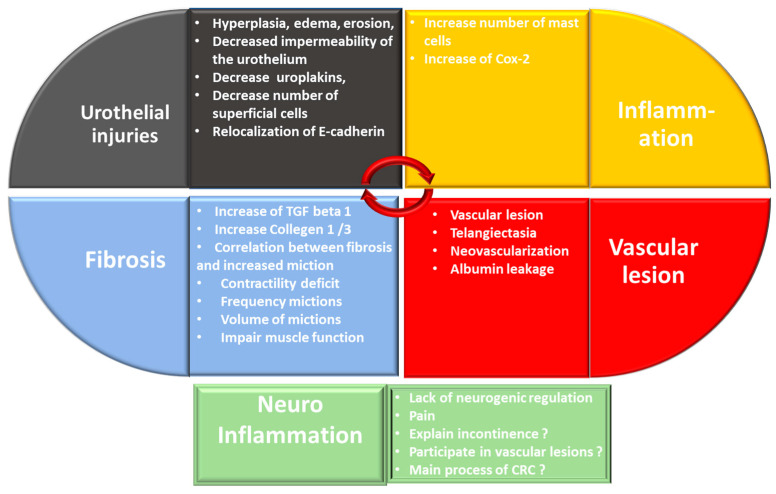
Synthesis of knowledge on the CRC and avenues for future study.

Excessive deposition of ECM impairs muscle function and muscle regeneration after irradiation. The mechanism by which profibrotic factors activate muscle fibrosis is an essential step toward developing anti-fibrotic treatments. According to Ikeda et al., the increase in collagen density and the invasion of the extracellular matrix into the muscles leads to a loss of muscle and bladder contractile capacity. The consequence is incomplete, sudden and involuntary bladder contractions [63]. Based on studies by Stewart et al. and Zwaans et al., the loss of contractility causes an increase in urinary frequency but no change in the volume of urine. The importance of incontinence increases with the irradiation dose, but not the time of initiation, which occurs between four months and one year after irradiation [49,51,53,54,64]. In the Stewart study, the increased urinary frequencies are correlated with a decrease in total bladder volume [49,51,53,54]. Fragmentation plays a very important role in the onset of symptoms. The higher the number of fractionations, the greater the total radiation dose must be to have the same physiological effect. Based on the Stewart et al. and Kolher et al. studies, fractionation has a protective role on the bladder’s capacities [54,68,74]. Further in-depth studies will therefore be needed to elucidate the mechanisms underlying these effects and to understand the possible implications of fibrosis on the loss of bladder contraction (Figure 7).

## 8. Conclusions

**Figure 8:**
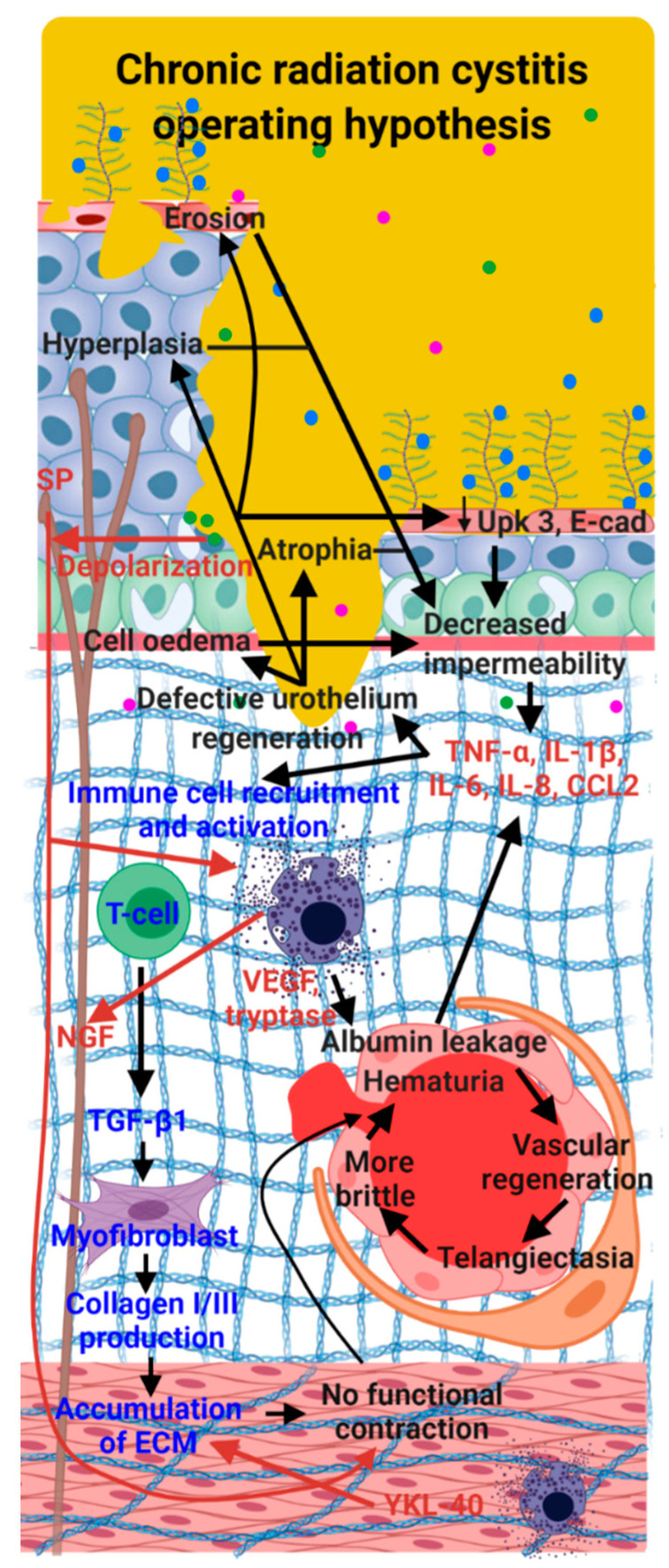
Hypothesis of CRC mechanisms in red. The first step is urothelium destruction mediating hyperplasia, cellular edema or atrophy. The secretion of inflammatory factors produced by urothelial injuries (TNF-α, IL1-β, IL-6, IL-8 and chemokine (C-C motif) ligand 2 (CCL2)) induces enrolment and stimulation of immune cells (mast cells). Mast cells secrete VEGF and tryptases, which induce vascular damage and imperfect vascular regeneration, telangiectasia, amplified vascular permeability, hematuria. This leads to the release of albumin. Albumin strengthens the secretion of pro-inflammatory factors which increase inflammation and may lead to the preservation of urothelial injuries. Mast cells, through the secretion of NGF, mediate the hyperactivation of nerve fibers. Secretion of TGF-β could mediate excessive deposition of ECM (collagen I and III, fibronectin) in the submucosa and smooth muscle. Black arrows indicate specific mechanisms of CRC, blue arrows designate shared mechanisms between CRC, interstitial and hemorrhagic cystitis; and red arrows specify hypotheses of how radiation cystitis may operate based on mechanisms of both interstitial and hemorrhagic cystitis.

The etiology of CRC is poorly understood and is mainly related to urothelium degradation and hemorrhage. The reason for this is that there is only a small number of preclinical studies on the subject, thus limiting our understanding of this pathology. Furthermore, the studies that do exist focus mainly on telangiectasia, fibrosis, hematuria and loss of bladder function. The molecular process remains poorly described in terms of inflammation and detrusor muscle damage. There is a need to deepen our knowledge in order to develop a better understanding of the CRC pathology and be able to propose innovative treatments (Figure 8).

One point that has been poorly studied is the role of the urothelium in initiating CRC. Imperfect repair could explain the latency phase observed between the acute and chronic phases. The urothelium could be the initial element in the onset of the chronic phase of radiation cystitis. During the chronic phase, structural abnormalities of the urothelium, such as hyperplasia and cellular edema, favor incomplete repair and a decrease in the impermeability of the urothelium, allowing urine to infiltrate the tissues, thus promoting inflammation. During CRC, the urothelium presents molecular abnormalities with a decrease in Uroplakine III (Upk3) and E-cadherin (E-cad), which results in a decrease in impermeability. The complex processes of deregulation of the homeostasis of the urothelium may induce hyperplasia, thinning and loss of impermeability. A study of the correlation over time of the multiple cellular lesions of the urothelium, the renewal of the urothelium and its impermeability together would be necessary to better understand this process. Overall, preclinical studies tend to agree on the vascular origin of the appearance of CRC. The lesions caused by irradiation and by reactive oxygen species (ROS) to epithelial cells are proportional to the irradiation dose [57] and release pro-inflammatory factors. Activation of mast cells by these pro-inflammatory factors leads to neo-vascularization and permeability of the blood vessels [82]. The consequence is a presence of albumin outside the vessels that promotes the recruitment and activation of new mast cells. These leakages maintain the inflammation, which becomes chronic. 

Inflammation induces significant fibrosis of the tissue (through the TGF and SMAD pathway) which invade the detrusor and the submucosa, particularly around the blood vessels, making them less “flexible”. This fibrosis causes a decrease in bladder capacity and difficulties in contractions leading to spasms, emergencies, etc. The spasms provoke the rupture of the vessels, inducing microscopic and then macroscopic hematuria, which favors inflammation. From then on, there will be a vicious circle with vascular damage producing inflammation, neuroinflammation, lack of neurogenic control promoting pain, fibrosis and vascular lesions. According to Steineck et al. [106], we might find new insights if we try to better understand which pathophysiological processes are triggered by radiotherapy at the organ level. For the irradiated bladder, is there a chronological sequence of pathophysiological events or a neurogenic lack of definitive controls leading to CRC?

## Figures and Tables

**Table 1:** Organs at risk according to the organ of the abdomino-pelvic sphere treated by radiotherapy.

Organ Treated by Radiotherapy	Organ at Risk
Rectum	Small intestine
Anal canal	Small intestine, bladder, vulva, labia majora
Cervix	Rectum, bladder, anal canal, small intestine, sigmoid colon, vagina
Endometrium	Rectum, bladder, anal canal, small intestine, sigmoid colon, vagina
Vulva	Rectum, bladder, urethra, anal canal, small intestine, sigmoid
Prostate	Rectum, bladder, anal canal
Bladder	Rectum, anal canal, small intestine, sigmoid colon
Testis	Spinal cord, kidney, stomach, small intestine, colon

**Table 2:** Classification systems for the severity of cystitis.

Grade	CTCAE v5.0 [33]	RTOG/EORTC [26]	LENT-SOMA [32]
I	Minimal or microscopic bleeding intervention not indicated	Slight epithelial atrophy Minor telangiectasia (microscopic hematuria)	Hematuria occasional and microscopic Dysuria occasional Incontinence weekly episodes
II	Gross bleeding, medical intervention or urinary tract irrigation indicated	Moderate frequency Generalized telangiectasia Intermittent macroscopic hematuria	Hematuria intermittent, macroscopic and <10% decrease hemoglobin Dysuria intermittent Incontinence daily episodes
III	Transfusion, interventional radiology, endoscopic or operative intervention indicated, radiation therapy (i.e., hemostasis of bleeding site)	Severe frequency and dysuria Severe generalized telangiectasia (often with petechiae) Frequent hematuria Reduction in bladder capacity (<150 cc)	Hematuria persistent, macroscopic and 10–20% decrease hemoglobin Dysuria persistent and intense Incontinence daily episodes
IV	Life-threatening consequences, major urgent intervention indicated	Necrosis/Contracted bladder (capacity <100 cc) Severe hemorrhagic cystitis	Hematuria persistent, >20% decrease hemoglobin Dysuria refractory and excruciating Incontinence complete refractory

Abbreviations: CTCAE = Common Terminology Criteria for Adverse Events; RTOG/EORTC = Toxicity criteria of the Radiation Oncology Group (RTOG)/European Organization for Research and Treatment of Cancer (EORTC); LENT-SOMA = Late Effects Normal Tissue (LENT)-Subjective Objective Management Analytic (SOMA).

**Table 3:** Summary of Pre-Clinical Studies of Chronic Radical Cystitis (1978 to 2021).

Model	Dose	Irradiation Source	Study Time	Main Characteristic Studied	Method Used	Main Results	Ref
CBA Female Mouse	Single dose from 10 to 40 Gy (5 Gy increments)	Linear accelerator (1.8 MeV, 75 pulses/sec). 112.5 Gy/min	Monthly (for 16 months)	Miction	Metabolic Cage	Increase in urinary frequency with the irradiation dose	[57]
CBA Female Mouse	Single dose from 10 to 40 Gy (5 Gy increments)	Linear accelerator (1.8 MeV, 75 pulses/sec). 112.5 Gy/min	3, 7, 9, 12, 19 and 22 months	Urothelium regeneration Endothelium regeneration	Tritium marking Tritium marking	Increase in urothelium regeneration speed between 6 and 22 months inversely proportional to the dose Dose-dependent increase in endothelium regeneration rate between 6 and 22 months.	[58]
CBA Female Mouse	Single or fraction dose: 2 (24 h) and 5 fractions (4 days) Single dose: 15 to 32.5 Gy (2.5 Gy increments) Dose for 2 fractions: 22.5 to 37.5 Gy (5 Gy increments)Dose for 5 fractions: 30 to 55 Gy (5 Gy increments)	Linear accelerator (1.8 MeV, 75 pulses/sec). 112.5 Gy/min	6 to 15 months (monthly)	Miction Bladder capacity	Metabolic Cage	Increase in urinary frequency with the irradiation dose, but decrease in urinary frequency with the number of fractions from 12 months onwards. Progressive reduction of bladder capacity proportional to dose and increase of capacity proportional to the number of fractionations starting at 12 months.	[59]
Female mouse CBA/Ht Gy f BSVS	Fractionation: 1, 5, 10 or 20 fractions (equal dose per fraction spread over 1 to 2 weeks (condition 20 fractions = 2 fractions per day separated by 5 h)) Irradiation dose for 1 fraction: 20, 25 and 30 Gy. For 5 fractions: 35, 45, 55 and 60 Gy. For 10 fractions: 45, 55, 65 and 75 Gy. For 20 fractions: 50 to 90 Gy (increment of 10 Gy).	Linear accelerator (1.8 MeV, 75 pulses/sec). 112.5 Gy/min	9, 10, 11, 13, 14 months	Miction Bladder capacity	Metabolic cage	Increase in urinary frequency with the irradiation dose, but decrease in urinary frequency with the number of fractions from 10 months onwards Progressive reduction of bladder capacity proportional to dose and increase of capacity proportional to the number of fractionations starting at 10 months.	[60]
Female mouse CBA/Ht Gy f BSVS	**Electron**: 1 fraction: 17.5, 22.5, 25, 30 Gy 2 fractions: 22.5, 27.5, 32.5, 37.5 Gy; 4 fractions: 35, 45, 55 Gy 8 fractions: 35, 45, 55, 65 Gy **Neutron**: 1 fraction: 7, 8, 9, 10 Gy 2 fractions: 7.5, 9, 11, 12.5 Gy 4 fractions: 8, 10, 12, 14 Gy 8 fractions: 8, 10, 12, 14, 16 Gy	1.8 MeV electron linear accelerator or 3 MeV neutron linear accelerator	9 to 14 months (monthly)	Miction	Metabolic cage	Increase in urinary frequency with the irradiation dose but decrease in urinary frequency with the number of fractions from 9 months onwards Irradiation with electron is less damaging.	[25]
Female mouse CBA/Ht Gy BSVS	Single dose from 15 to 32.5 (2.5 Gy increments)	Linear accelerator (1.8 MeV, 75 pulses/sec). 112.5 Gy/min	16 months (monthly)	Miction Urothelium regeneration Endothelium regeneration	Metabolic cage Tritium marking Tritium marking	Increase in urinary frequency from 7 months and proportional to the irradiation dose Limited urothelium regeneration in the first 3 months after irradiation. Limited regeneration of the endothelium in the first 3 months after irradiation.	[61]
Female mouse C3H/Hen Af-nu+	Single dose of 10, 15, 20, 25, 27.5 and 30 Gy	X-rays 250 kV, filtered at 0.5 mm Cu, at 15 mA. At 2.1 Gy/min	Day 5, 12, 19 then every 4 to 6 weeks (over 53 weeks)	Urinary frequency Max. bladder volume Urothelium	Metabolic cage Cystoscopy Histology	Increase in urinary frequency as a function of time (acute phase in the 1st month) and proportional to the dose. Decrease in max. bladder volume as a function of time (acute phase in the 1st month) and proportional to the dose Epithelial denudation and focal hyperplasia, with fibrosis and ulceration.	[62]
New Zealand male rabbit	Fractionated doses over 5 consecutive days (once a day) for a total of 33, 36 and 39 Gy. Irradiation of the entire bladder or half of the bladder on the cranial or caudal side.	X-ray of 300 kV 0.8 Gy/min (Seifert isovolt 3002; filtration: 1.0 mm Al)	100 weeks	Miction	Metabolic cage	Increase in frequency of urination decrease in max. volume for 100 weeks for dose 39 Gy or 36 Gy (total bladder irradiation), and from 20 weeks onwards for irradiation on half of the bladder (39 Gy at the cranial part of the bladder and 39 Gy or 36 Gy at the caudal part)	[63]
New Zealand male rabbit	Fractionated doses over 5 consecutive days (once a day) for a total of 33, 36 and 39 Gy. Irradiation of the entire bladder or half of the bladder on the cranial or caudal side.	X-ray of 300 kV 0.8 Gy/min (Seifert isovolt 3002; filtration: 1.0 mm Al)	100 weeks	Tissue alteration	Histology	Dose-dependent urothelial hyperplasia or atrophy of the urothelium and more pronounced in the body of the bladder and trigone, submucosal and muscular tissues showed dose-dependent fibrosis and changes in the blood and lymphatic vessels, the body and trigone are more sensitive to radiation (more fibrosis, changes in the vessels)	[64]
Female mouse C3H/Hen Af-nu+	Single dose of 10, 15, 20, 22.5, and 25 Gy	X-ray 250 kV, filtered at 0.5 mm Cu, at 15 mA, 2.1 Gy/min	1, 2 and 4 weeks after irradiation then monthly up to 61 weeks (irradiation in week 4 or 12)	Urinary frequency Volume per voiding Max. bladder volume	Absorbent paper (15 cm/h) Scanning tablet (Calcomp B.V., Amstelveen, The Netherlands) with special program Cystoscopy	Increased frequency in acute phase during the first 4 weeks, and late phase from 15 to 37 weeks (dose-dependent). Volume reduction per voiding between 52 and 56 weeks for 25 Gy. Decrease in total bladder volume as a function of the dose.	[65]
Female mouse C3H/Neu Female mouse C3H/Hen Af-nu+	Single dose of 19 Gy Single dose of 25 Gy and 14 Gy.	Seifert Isovolt 320 300 kV, 5.3 Gy/min Seifert Isovolt 320 250 kV 2.1 Gy/min	90 to 360 days	Bladder function Fibrosis Urothelium	Cystoscopy Immunohistochemistry Histology	Correlation between decrease in max. bladder volume and the expression of TGFβ and collagen, decrease in total bladder volume Increased tissue protein expression in TGFβ (in both strains) as a function of time and dose, increased collagen I and III from 180 days after irradiation, but decreased collagen I/III ratio in C3H/Hen Af-nu+ mice and increased in CH3/Neu mice Increased denudation or hyperplasia as a function of the dose, edema.	[66]
Female mouse C3H/Neu	Single dose of 20 Gy	Seifert Isovolt 320/20 X-ray machine (Seifert X-ray Corp., Fairview Village, PA) 200 kV, 0.85 Gy/min	2, 4, 7, 10, 13, 16, 19, 22, 25, 28, 31 days (early), then on days 90, 120, 180, 240 and 360 (late) after irradiation.	Vascular	Immunohistochemistry	Increased COX-2 expression in the blood vessel wall during the precocious phase (4 to 16 days) but not in the late phase.	[67]
Female mouse C3H/Neu	Single dose of 20 Gy	Seifert Isovolt 320/20 X-ray machine (Seifert X-ray Corp., Fairview Village, PA) 200 kV, 0.85 Gy/min	2, 4, 7, 10, 13, 16, 19, 22, 25, 28, 31 days (early), then on days 90, 120, 180, 240 and 360 (late) after irradiation.	Urothelium	Immunohistochemistry	Decrease in superficial cells in early phase (day 0 to 31) until the beginning of late phase (90 to 120 days), progressive loss of Uroplakine III expression at the cell surface correlated with the loss of superficial cells, but increase in cytoplasmic expression of Uroplakine III in superficial cells until the beginning of late phase (120 days)	[68]
Female mouse C3H/Neu	Single dose of 20 Gy	Seifert Isovolt 320/20 X-ray machine (Seifert X-ray Corp., Fairview Village, PA) 200 kV, 0.85 Gy/min	90, 120, 180, 240 and 360 (late response phase) after irradiation	Fibrosis Vascular	Histology Immunohistology	Infiltration of albumin around small blood vessels in early and late phase Increase in collagen deposition in the bladder.	[69]
Lewis Rats female	Single dose of 20 Gy	Cesium irradiation (about 4 Gy/min)	1.5 and 3 months	Fibrosis Vascular lesion	Histology Histology and transcriptomic	Increase in collagen at 3 months in muscle with fibrosis and overexpression of the TGF β gene at 3 months. Decrease in the number of blood vessels at 1.5 and 3 months, preferably in the dome, without increasing the expression of the VEGF gene.	[70]
Female mouse C3H/HeN	Single dose of 20 Gy	Small Animal Radiation Research Platform (SARRP), 2 Gy/min 220 kV and 13 mA	19 weeks	Miction Fibrosis Urothelium Inflammation	Metabolic cage Histology, Immunohistology Histology	Increase in urinary frequency with decrease in volume per void. Increased fibrosis Decreased Upk III, E-cadherin and CD31 expression, hyperplasia. Increase of mast cells	[20]
Female mouse C57Bl/6	Single dose of 10 Gy, external bladder	X-RAD 320 biological irradiator (Precision X-Ray, North Branford, CT), dose rate not given	1, 2, 4, 6 and 9 and 12 weeks	Miction Bladder contraction Fibrosis	Whatman filter paper Electromyogram Histology	Increase in urinary frequency/incontinence 9 weeks after irradiation Decrease in contractility and compliance, increase in passive tension 9 weeks after irradiation. Increased collagen deposition (changes wall relaxation).	[71]
Female Mouse C57BL/6, C3H and BALB/c	Single dose of 40 Gy	Small Animal Radiation Research Platform (SARRP), 2 Gy/min 220 kV et 13 mA	12 months	Miction Fibrosis Vascular	Metabolic Cage Histology, immunohistology Photos	Increased urinary frequency with decreased volume per void in C57BL/6 Increased fibrosis, collagen densification in C57BL/6 and BALB/c, increase in collagen I and III than in C57BL/6. Pale avascular lesions, hemorrhages and telangiectasias.	[15]

## Data Availability

Not applicable.
